# Chemical shift assignments of retinal guanylyl cyclase activating protein 5 (GCAP5) with a mutation (R22A) that abolishes dimerization and enhances cyclase activation

**DOI:** 10.1007/s12104-023-10129-3

**Published:** 2023-05-02

**Authors:** Diana Cudia, Effibe O. Ahoulou, James B. Ames

**Affiliations:** Department of Chemistry, University of California, Davis, CA 95616 USA

**Keywords:** Retinal guanylyl cyclase, GCAP5, R22A, EF-hand, Phototransduction

## Abstract

Retinal membrane guanylyl cyclases (RetGCs) in vertebrate rod and cone photoreceptors are activated by a family of neuronal Ca^2+^ sensor proteins called guanylyl cyclase activating proteins (GCAP1-7). GCAP5 from zebrafish photoreceptors binds to RetGC and confers Ca^2+^/Fe^2+^-dependent regulation of RetGC enzymatic activity that promotes the recovery phase of visual phototransduction. We report NMR chemical shift assignments of GCAP5 with a R22A mutation (called GCAP5^R22A^) that abolishes protein dimerization and activates RetGC with 3-fold higher activity than that of wild type GCAP5 (BMRB No. 51,783).

## Biological context

Guanylyl cyclase activating proteins (GCAP1-7) are Ca^2+^-binding proteins in the retina that belong to a sub-branch of the calmodulin superfamily (Burgoyne 2007, Lim, Dizhoor et al., 2014). GCAP proteins contain an N-terminal myristoyl group and four EF-hand motifs that bind to Ca^2+^ at EF2, EF3 and EF4 (Ames 2021). The first EF-hand contains residues that disable Ca^2+^ binding and the Ca^2+^-free EF1 interacts with the N-terminal myristoyl group (Cudia, Roseman et al., 2021, Stephen, Bereta et al., 2007). The Ca^2+^-bound GCAPs bind to RetGC and inhibit its cyclase activity, whereas Ca^2+^-free GCAPs activate RetGC enzymatic activity during the recovery phase of visual phototransduction (Koch and Stryer 1988, Palczewski, Subbaraya et al., 1994, Peshenko and Dizhoor 2007). Light activation of retinal photoreceptor cells causes a decrease in the cytosolic Ca^2+^ concentration that serves as a coordinating signal for visual recovery (Arshavsky and Burns 2014). The light-induced drop in cytosolic Ca^2+^ concentration is sensed by GCAPs that promote Ca^2+^-sensitive activation of RetGC to replenish cGMP levels in order to restore the dark state (Koch and Helten 2008; Koch and Stryer 1988). Mutations in GCAP1 that weaken Ca^2+^ binding or otherwise alter Ca^2+^-sensitive activation of RetGC are genetically linked to retinal diseases (Jiang and Baehr 2010, Payne, Downes et al., 1998).

GCAP5 in zebrafish photoreceptors binds to both Ca^2+^ and Fe^2+^ (Lim et al. 2017). The Ca^2+^-free forms of GCAP1 (Peshenko and Dizhoor 2006) and GCAP5 (Lim et al. 2017) both activate RetGC activity in light-adapted photoreceptors, whereas the Ca^2+^-bound GCAP1 (Peshenko and Dizhoor 2007) and Fe^2+^-bound GCAP5 (Lim et al. 2017) both inhibit RetGC in dark-adapted photoreceptors. The NMR structure of GCAP5 (Cudia et al. 2021) revealed that GCAP5 forms a dimer in solution with key amino acid residues at the dimer interface (H18, Y21, R22, M25, F72, V76 and W93) that are important for cyclase activation. The GCAP5 mutations H18E, M25E and V76E each abolish GCAP5 dimerization and prevent activation of RetGC (Cudia et al. 2021). These results suggested that GCAP5 dimerization might be essential for RetGC activation (Ames 2021, 2022). However, this hypothesis was refuted by the discovery that the R22A mutation of GCAP5 not only abolishes GCAP5 dimerization but also causes a 300% increase in RetGC activation compared to that of wild type (Cudia et al. 2021). We hypothesize that the R22A mutation might somehow alter the structure of GCAP5 to abolish its dimerization and increase its potency for activating RetGC. We report here NMR resonance assignments for the Ca^2+^-free activator and monomeric form of Ca^2+^-free GCAP5 with the R22A mutation (called GCAP5^R22A^) to understand how this mutation abolishes protein dimerization and causes a 300% increase of RetGC activity compared to that of wild type GCAP5.

## Methods and experiments

### Preparation of GCAP5

Samples of recombinant myristoylated GCAP5^R22A^ (residues 2-198) uniformly labeled with ^15^ N and ^13^ C were prepared as described previously for wild type GCAP5 (Cudia and Ames 2019; Cudia et al. 2021).

### NMR spectroscopy

NMR samples of Ca^2+^-free and myristoylated GCAP5^R22A^ were prepared as described previously for wild type GCAP5 (Cudia et al. 2021). The NMR samples consisted of 0.3 mM protein dissolved in 5 mM TRIS-d_11_ (pH 7.4), 2 mM DTT-d_10_, 1 mM EDTA, 1 mM EGTA, 0.04% w/v NaN_3_, and 92% H_2_O/7% D_2_O. All NMR experiments were performed at 32 °C on a Bruker Avance 600 MHz spectrometer equipped with a triple resonance cryogenic (TCI probe) as described previously (Cudia and Ames 2019). The following 3D NMR experiments (HNCA, HNCACB, HNCOCACB, HNCO, HBHACONH, and HBHANH) were analyzed to obtain backbone assignments (Ikura, Kay et al., 1990). Side chain resonances were assigned by analyzing HBCBCGCDHD, HBCBCGCDHDCEHE, and HCCH-TOCSY (Ikura, Spera et al., 1991). The software NMRPipe (Delaglio, Grzesiek et al., 1995) was used to process all NMR data, and Sparky NMRFAM (Lee, Tonelli et al., 2015) was used to obtain resonance assignments.

## Extent of assignments and data deposition

**Fig. 1:**
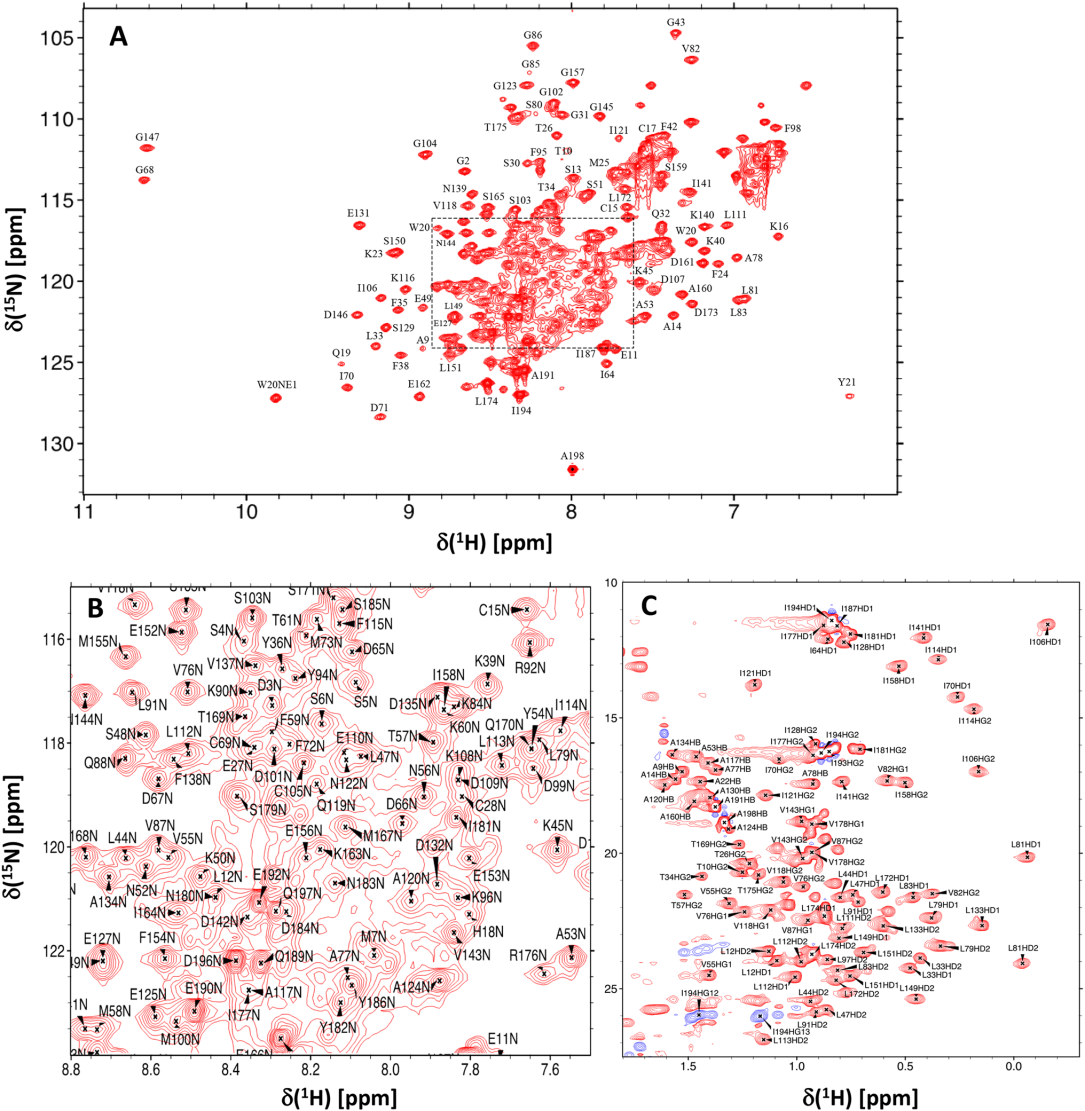
**A** Two-dimensional ^15^ N-^1^ H HSQC spectrum of ^15^ N-labeled Ca^2+^-free GCAP5^R22A^ illustrates backbone amide assignments. **B** Expanded view of the spectrally crowded central region surrounded by a box in panel A. **C** Constant-time ^13^ C-^1^ H HSQC spectrum of ^13^ C-labeled Ca^2+^-free GCAP5R22A illustrates side-chain methyl assignments indicated by residue labels

Representative NMR assignments are illustrated by two-dimensional NMR spectra of Ca^2+^-free GCAP5^R22A^ (^15^ N-^1^ H HSQC, Fig. 1A-B and ^13^ C-^1^ H HSQC, Fig. 1C). The resonance assignments were determined by analyzing 3D triple resonance NMR spectra of ^13^ C/^15^ N-labeled GCAP5^R22A^. The highly resolved NMR peaks with uniform intensities indicate a stable and folded structure. Amide resonances assigned to Q19, L33 and I70 exhibited noteworthy downfield shifts, perhaps because these residues are flanked by nearby aromatic rings (W20, F35 and F72 respectively) (Fig. 1A). The amide resonances assigned to G68 and G147 have downfield chemical shifts that are caused by a strong hydrogen bond between the backbone NH of G68 (EF2)/G147 (EF4) with side chain carboxyl groups of D63 (EF2)/D142 (EF4), respectively. These strong hydrogen bonds are stabilized by an open conformation for both EF2 and EF4. It is unusual for Ca^2+^-free EF-hands to occupy an open conformation that is typically only formed by Ca^2+^-bound EF-hands (Ikura 1996, Yap, Ames et al., 1999). However, the NMR structure of wild type Ca^2+^-free GCAP5 revealed that the Ca^2+^-free structures of EF2, EF3 and EF4 each adopt a pre-formed open conformation (Cudia et al. 2021), which might explain why the GCAP proteins exhibit such high affinity Ca^2+^ binding in the nanomolar range (Lim, Peshenko et al., 2009). Spectral assignments were obtained for more than 94% of the main chain ^13^ C resonances (^13^Cα, ^13^Cβ, and ^13^CO), 97% of non-proline backbone amide resonances (^1^HN, ^15^ N), and 87% of side chain resonances (Fig. 1C). The unassigned residues (A22, N46, E74, Y75, and I136) had weak HSQC peaks caused by exchange broadening that prevented their assignment. Complete chemical shift assignments (^1^H, ^15^N, ^13^C) of Ca^2+^-free GCAP5^R22A^ have been deposited in the BioMagResBank (http://www.bmrb.wisc.edu) under accession number 51,783.

**Fig. 2:**
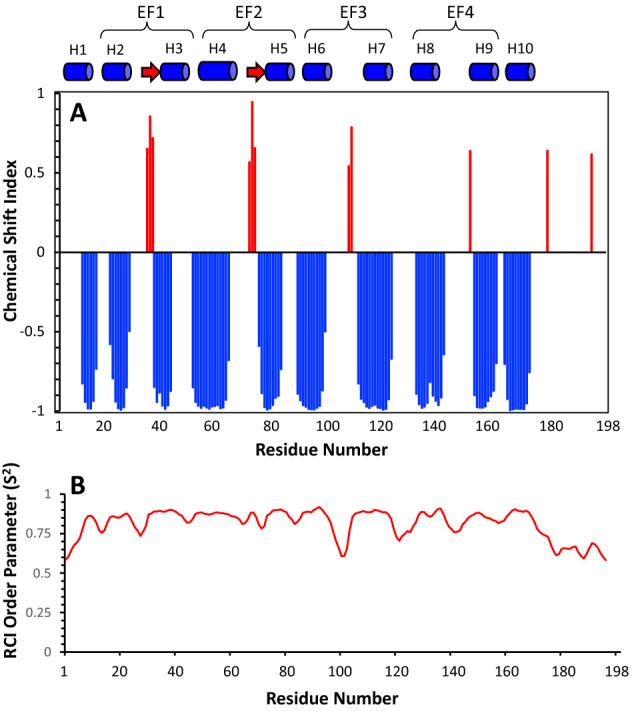
Secondary structure and RCI order parameter (*S*^2^) of Ca^2+^-free GCAP5^R22A^ predicted from the assigned backbone chemical shifts. **A** Probability of secondary structural elements (blue cylinders for helix and red arrow for strand) and **B** RCI *S*^2^ of Ca^2+^-free GCAP5^R22A^ were calculated using TALOS + server (Shen et al. 2009)

Chemical shift index (Wishart, Sykes et al., 1992) and secondary structure prediction software using TALOS+ (Shen, Delaglio et al., 2009) were both used to calculate the secondary structure of Ca^2+^-free GCAP5^R22A^ (Fig. 2A, B). GCAP5^R22A^ has the same secondary structure that was reported previously for wild type GCAP5 (Cudia and Ames 2019): The protein has 10 α-helices: H1 (residues 8–14), H2 (residues 18–26), H3 (residues 35–41), H4 (residues 49–62), H5 (residues 74–82), H6 (residues 87–95), H7 (residues 110–117), H8 (residues 129–135), H9 (residues 150–160) and H10 (residues 162–172) shown as cylinders in Fig. 2B. Helices H2–H9 form four EF-hand motifs as seen in previous structures of GCAP1 (Lim, Peshenko et al., 2016, Stephen et al. 2007) and GCAP5 (Cudia et al. 2021). A 3-residue β-strand is observed in the Ca^2+^-free binding loops of EF1 and EF2 (shown as red arrows in Fig. 2A). This β-strand is only partially formed in the third and fourth EF-hands of Ca^2+^-free GCAP5. The final 14 residues from the C-terminus in GCAP5^R22A^ (residues 184–198) are dynamically disordered and unstructured like was seen in previous structures of wild type GCAP5 (Cudia et al. 2021) and GCAP1 (Stephen et al. 2007).

**Fig. 3:**
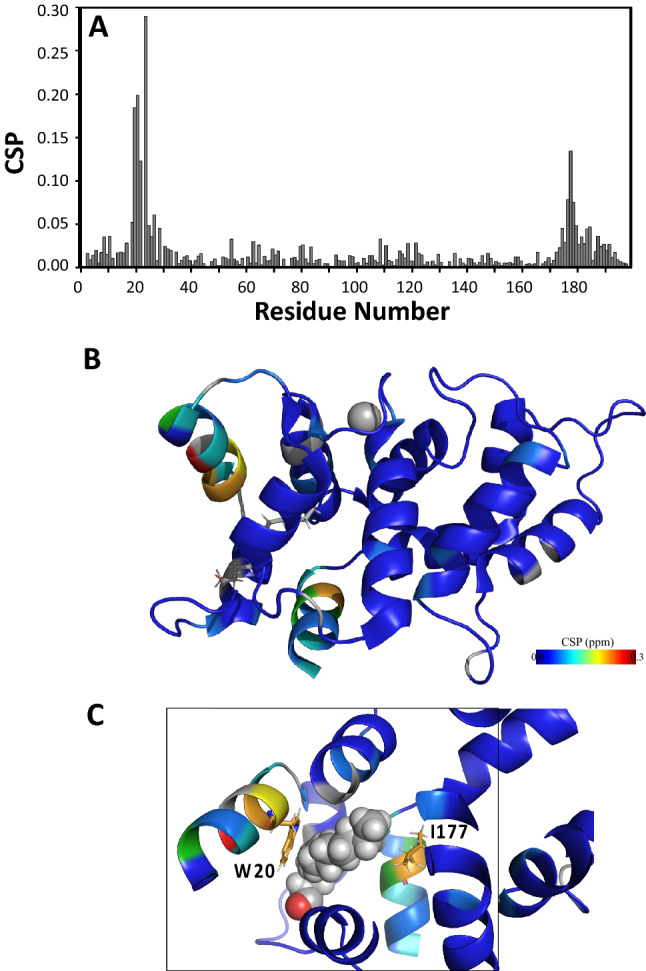
Chemical shift perturbation (CSP) for Ca^2+^-free GCAP5^R22A^ versus wild type GCAP5. **A** Backbone amide CSP was calculated as: $CSP= \sqrt{{\left(\varDelta {H}^{N}\right)}^{2}+{\left(0.14\times \varDelta N\right)}^{2}}$. ΔH^N^ and ΔN are the observed difference in the ^1^H^N^ and ^15^ N chemical shifts, respectively between Ca^2+^-free GCAP5^R22A^ (BMRB 51,783) and wild type GCAP5 (BMRB 51,783). **B** CSPs mapped on the structure of GCAP5 (Cudia et al. 2021). **C** Close-up view of the myristoyl group binding site environment in GCAP5. The side chains of W20 and I177 make close contacts with the myristoyl group

The assigned amide chemical shifts of Ca^2+^-free GCAP5^R22A^ (BMRB 51,783) are compared to those of Ca^2+^-free GCAP5 wild type (BMRB 51,784) to help identify residues that are structurally affected by the R22A mutation (Fig. 3A). Not surprisingly, the GCAP5 residues in EF1 (Q19, W20, Y21 and K23) that are closest to R22A exhibit the largest chemical shift perturbation (Fig. 3A, B). In addition, C-terminal residues R176, I177 and V178 also exhibit detectably large chemical shift perturbations. In the wild type GCAP5 structure (Cudia et al. 2021), the side-chain methyl groups of I177 are in close proximity with the side chain indole group of W20, and both side chains make close contact with the N-terminal myristoyl group (Fig. 3C). Interestingly, the myristoyl group contacts with both W20 and I177 are both important for the proposed Ca^2+^-myristoyl tug mechanism that transmits Ca^2+^-induced conformational changes from the EF-hands to the myristoyl group (Peshenko, Olshevskaya et al., 2012). We suggest that the R22A mutation may stabilize the Ca^2+^-free GCAP5 activator conformation by disrupting the Ca^2+^-myristoyl tug (Peshenko et al. 2012). The NMR assignments of Ca^2+^-free GCAP5^R22A^ presented here suggest the R22A mutation affects the structure in both EF1 (W20) and C-terminal region (I177) that may play a role in disrupting GCAP5 dimerization and enhancing cyclase activation.

## Data Availability

The assignments have been deposited to the BMRB under the accession code: 51,783.
